# Changes in biomass allocation buffer low CO_2_ effects on tree growth during the last glaciation

**DOI:** 10.1038/srep43087

**Published:** 2017-02-24

**Authors:** Guangqi Li, Laci M. Gerhart, Sandy P. Harrison, Joy K. Ward, John M. Harris, I. Colin Prentice

**Affiliations:** 1 Department of Biological Sciences, Macquarie University, North Ryde, NSW 2109, Australia; 2 School of Archaeology, Geography and Environmental Sciences (SAGES), Reading University, Reading, UK; 3 Geography Department, Kansas State University, Manhattan, KS 66505, USA; 4 Department of Ecology and Evolutionary Biology, University of Kansas, Lawrence, KS 66045, USA; 5 The La Brea Tar Pits Museum (George C. Page Museum), 5801 Wilshire Boulevard, Los Angeles, CA 90036, USA; 6 AXA Chair of Biosphere and Climate Impacts, Department of Life Sciences, Imperial College London, Silwood Park Campus, Buckhurst Road, Ascot SL5 7PY, UK

## Abstract

Isotopic measurements on junipers growing in southern California during the last glacial, when the ambient atmospheric [CO_2_] (c_a_) was ~180 ppm, show the leaf-internal [CO_2_] (c_i_) was approaching the modern CO_2_ compensation point for C_3_ plants. Despite this, stem growth rates were similar to today. Using a coupled light-use efficiency and tree growth model, we show that it is possible to maintain a stable c_i_/c_a_ ratio because both vapour pressure deficit and temperature were decreased under glacial conditions at La Brea, and these have compensating effects on the c_i_/c_a_ ratio. Reduced photorespiration at lower temperatures would partly mitigate the effect of low c_i_ on gross primary production, but maintenance of present-day radial growth also requires a ~27% reduction in the ratio of fine root mass to leaf area. Such a shift was possible due to reduced drought stress under glacial conditions at La Brea. The necessity for changes in allocation in response to changes in [CO_2_] is consistent with increased below-ground allocation, and the apparent homoeostasis of radial growth, as c_a_ increases today.

Fossil *Juniperus* spp. wood specimens from the La Brea Tar Pits in southern California (34.06°N, 118.36°W, 80 m a.s.l.) have been radiocarbon-dated to the second half of the last glaciation, between 55–22 ka BP, an interval when the climate was globally ca 5–8 °C colder than today^1^ and atmospheric CO_2_ concentration [CO_2_] was between 180–220 ppm^2^. Stable carbon isotope discrimination was measured on these specimens to estimate the ratio of leaf intercellular [CO_2_] (c_i_) to ambient [CO_2_] (c_a_) at the time of growth^3^. The c_i_/c_a_ ratio during glacial times was similar to that found today^3^,^4^, implying c_i_ values of only 100–120 ppm (i.e. not far above the modern compensation point for C_3_ plants, ~40–70 ppm). The low c_i_ values imply a strong reduction in gross primary production (GPP). Nevertheless, remarkably, measurements of annual growth rate of these trees (as shown by the width of the annual rings) show that stem growth was similar to today^5^.

Palaeoenvironmental evidence indicates that the climate in the La Brea region during the glacial was both cooler and wetter than today^6^,^7^. Pollen-based reconstructions show a cooling of 2–6 °C in both summer and winter^8^. Mean annual precipitation was 100–300 mm more than today, as a result of circulation changes due to southward deflection of the Westerlies by the Laurentide Ice Sheet^9^,^10^,^11^, and relative humidity was also greater than present as shown using ^18^O data^3^. These changes in climate could potentially have compensated for the impact of c_i_ on GPP and growth, through the effect of lower temperature in reducing photorespiration (thus lowering the compensation point), and/or through reducing the potential loss of photosynthetic activity due to drought stress. However, as shown experimentally in herbaceous C_3_ species^12^,^13^, it is unlikely that these effects would have been sufficient to compensate for the reduction in GPP due to low c_i_.

A growing body of evidence suggests that changing [CO_2_] results in changes in carbon allocation between aboveground (leaf, stem) and underground (root) biomass pools. Observations of the response to artificially high [CO_2_] conditions in Free-Air Carbon Enrichment (FACE) experiments show that trees typically allocate more carbon below ground, in the form of increased root mass and increased exudates^14^,^15^,^16^,^17^,^18^,^19^,^20^, often at the expense of stem growth^21^. The widespread failure to detect a response to increasing [CO_2_] during the 20^th^ century in many tree-ring records^22^,^23^,^24^ is consistent with the idea that increased productivity due to increased [CO_2_] does not necessarily lead to increased stem growth and may be reflected in changes in allocation. Furthermore, there is some evidence for structural changes and decreased below ground allocation in modern C_3_ woody species grown at glacial [CO_2_]^25^,^26^.

Here we use a generic light-use efficiency model, ‘P’^27^,^28^ coupled to a species-specific carbon allocation model, ‘T’^29^ that has been shown to reproduce the observed growth response to climate and [CO_2_] changes during the historic period in both cold, humid and warm semi-arid conditions^29^,^30^, to investigate whether climate conditions and/or changes in allocation strategy facilitated the growth of junipers during the glacial at the La Brea site.

## Results

The average c_i_/c_a_ ratio of the fossil wood samples from La Brea dated to the glacial *sensu stricto* (55–22 ka BP) is 0.51 ± 0.02 (mean ± standard deviation), while the value for the sample closest to the glacial maximum is 0.53 ± 0.01 (22 ka BP sample), which is comparable to the value of 0.53 ± 0.05 for modern samples from six southern Californian sites^3^,^4^. The modern comparison sites are at higher elevations (630 to 2830 m a.s.l), at locations that are more similar in climate to the glacial climate of La Brea. The average ring width for all the fossil glacial specimens is 1.39 ± 0.9 mm while the value for the 22 ka BP sample is 1.83 ± 0.6 mm, which can be compared to 1.16 ± 0.8 mm for the modern trees^4^.

**Figure 1:**
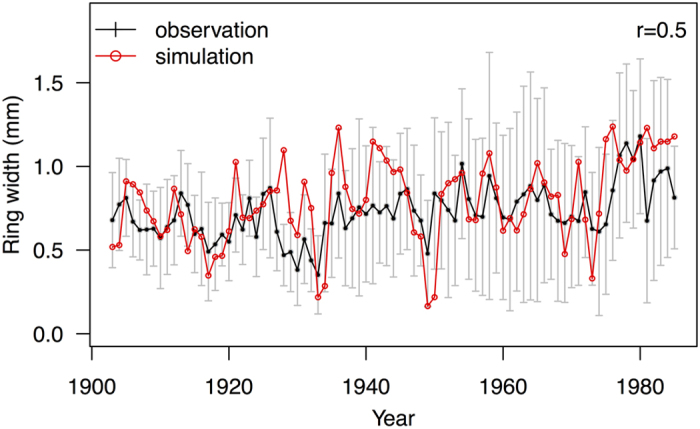
Evaluation of simulated and observed tree ring variations under modern climate conditions. Comparison between simulated and observed *Juniperus occidentalis* ring widths, for the period 1903 to 1985, from site CA640 (36.95°N, 118.92°W, 2630 m a.s.l.). The black line is the mean of observations and the grey bars are the standard deviation (SD) between trees. The red line is the mean from the simulations.

The six modern southern Californian records were primarily collected for isotopic measurements; some of these records are very short (<50 years) and there are too few replicates to provide a well-founded record of the response of stem growth to climate variability. To test how well the coupled light-use efficiency and carbon allocation model (hereafter called PT) explicitly simulates the radial growth of *Juniperus*, we used a record of *Juniperus occidentalis* from site CA640 (36.95°N, 118.92°W, 2630 m a.s.l.). CA640 provides a cross-dated record of tree ring widths for the period 1903 to 1985^31^; however, there are no isotopic measurements from this record. The PT model captures the observed amplitude and interannual variability of radial growth at site CA640 during the historic period (Fig. 1). The simulated mean ring width is 0.79 mm compared to the observed average width of 0.73 mm, and the correlation between simulated and observed interannual variability is significant (*r* = 0.50, *p* < 0.001). The model captures longer-term trends (e.g. decadal trends) in growth better than interannual variability (for 10-year smoothed means, *r* = 0.8, *p* = 0.018). The simulations and observations show consistent responses to different climate drivers (Supplementary Fig. 1). These include a significant positive correlation with photosynthetically active radiation (PAR) and [CO_2_], a positive response to soil moisture (as measured by the ratio of actual to equilibrium evapotranspiration: α), a positive response to temperature, and a negative response to vapour pressure deficit (VPD). The response to temperature, α and VPD is significant in the simulations but not in the observations, probably because these relationships are obscured by local factors in the field environment.

**Figure 2:**
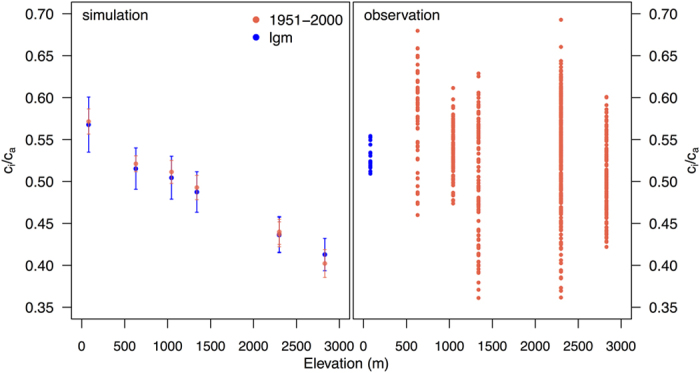
Changes in c_i_/c_a_ with elevation. The left panel shows the simulated c_i_:c_a_ at La Brea and the southern Californian sites under modern (red) and glacial (blue) conditions. The bars show two standard deviations from the mean value for each site during the simulation period (50 years for modern, 100 years for glacial). The right panel shows the observed c_i_:c_a_ from modern trees (all values for the period 1951–2000, red) and the 22 ka BP fossil sample from La Brea (blue).

Simulated c_i_ is obtained from c_a_ via the “least-cost hypothesis”^32^,^33^. The simulated c_i_/c_a_ ratio decreases with elevation under modern conditions at La Brea and the southern Californian sites (Fig. 2). This decline is partly a result of differences in temperature, which decreases from 16.6 °C at the lowest elevation to 6.7 °C at the highest elevation, and partly due to effects of decreasing air pressure. The simulated glacial c_i_/c_a_ ratio at La Brea and each of the modern southern Californian sites, derived using climate variables for the Last Glacial Maximum (LGM, ca 21,000 years ago) from the Community Climate System Model Version 4 (CCSM4) climate model^34^,^35^ adjusted to account for the bias in the modern control simulation and interpolated to the appropriate elevation, is the same as simulated under modern conditions (Fig. 2). The LGM c_i_/c_a_ simulated at La Brea is 0.57 ± 0.01, close to the value of 0.53 ± 0.01 obtained for the glacial sample closest to the LGM (22 ka BP).

**Figure 3:**
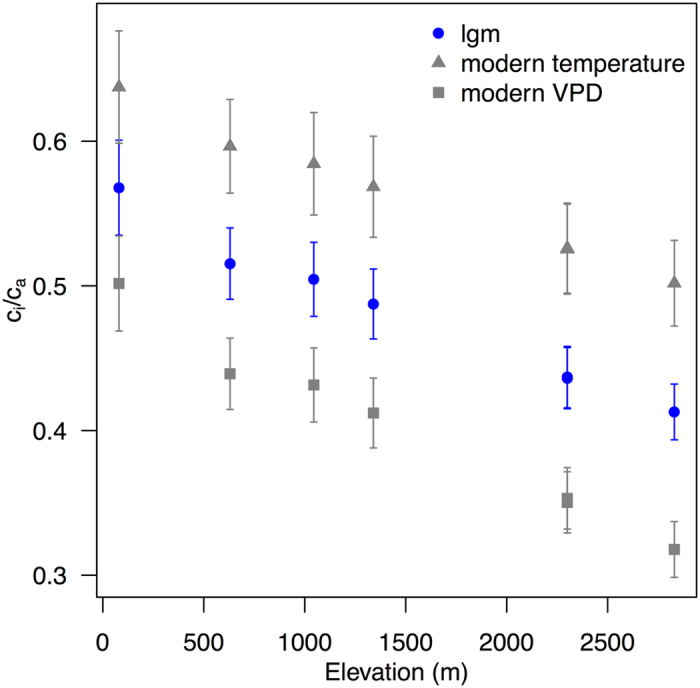
Impact of temperature and vapour pressure deficit (VPD) on c_i_/c_a_. The plot shows the simulated impact of using either modern-day temperature or modern-day vapour pressure deficit (VPD) on c_i_/c_a_ when all other climate variables are kept at glacial values. The blue symbols show the c c_i_/c_a_ ratio under full glacial conditions.

The known changes in regional climate at the LGM, specifically the year-round reduction in temperature and the decrease in aridity, would have opposite effects on c_i_/c_a_. According to the least-cost hypothesis (supported by field measurements in contrasting climates^33^ and a large global compilation of δ^13^C data^28^), the effect of a decrease in temperature is to increase water viscosity and hence the cost of water transport and reduce the cost of CO_2_ fixation by decreasing photorespiration, both of which should result in reduced c_i_/c_a_^28^. Conversely, a wetter climate would imply reduced VPD, which should tend to increase c_i_/c_a_. Sensitivity tests, using either modern temperature with glacial VPD or glacial temperature with modern VPD, show that these two effects are of roughly equal magnitude (Fig. 3).

The low glacial c_i_ values (simulated values ~90–110 ppm) result in simulated values of potential GPP of 3.9 kg C m^−2^ a^−1^. The simulated GPP at La Brea under modern climate is 4.3 kg C m^−2^ a^−1^, while simulated GPP under modern climate at the six higher-elevation sites ranges from 4.4 to 5.2 kg C m^−2^ a^−1^. To investigate this, we ran a suite of PT model simulations with glacial climate conditions and with [CO_2_] levels varying between 320 and 160 ppm. We used approximate Bayesian computation^36^,^37^,^38^ to determine what changes in the carbon allocation parameters (specifically leaf area index and the ratio of fine root mass to foliage area) would be required to maintain the observed ring width of 1.83 mm at 22 ka BP, keeping all other T-model parameters the same as in the modern simulations of CA640.

**Figure 4:**
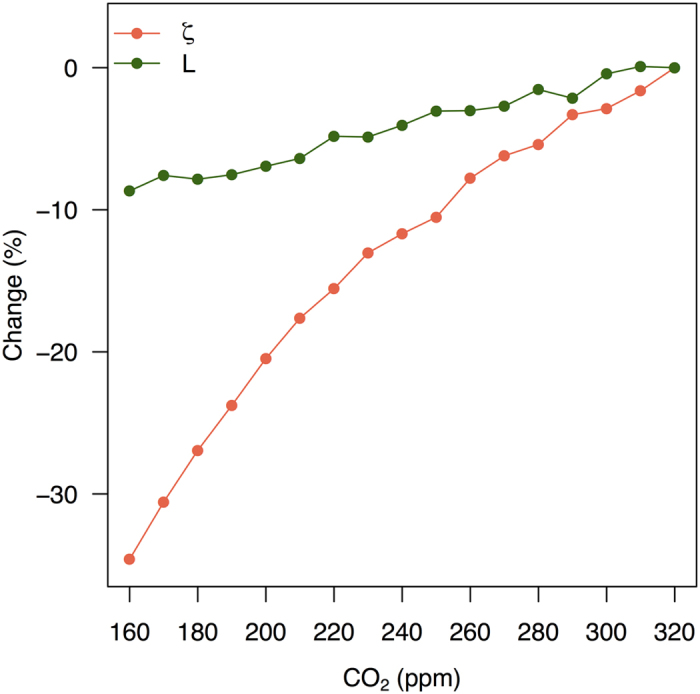
The impact of [CO_2_] on carbon allocation. The results show the impact of Bayesian optimisation using glacial climate variables and different levels of [CO_2_] between 320 and 160 ppm. The optimised variables are the ratio of fine-root mass to foliage area (*ζ*) and the leaf area index within the crown (L).

These simulations (Fig. 4) show that ring width can be maintained under glacial conditions by a small decrease in leaf area index, L, and by decreasing the ratio of fine root mass to foliage area, ζ. At 180 ppm (i.e. the approximate level of CO_2_ at 22 ka BP), the reduction in L is ca 8% and the reduction in ζ is ca 27% compared to values obtained with [CO_2_] levels corresponding to the mean during the 20^th^ century (320 ppm). Simulated photosynthetic capacity at a standard temperature of 25° C (V_cmax_[25]) was higher by 73% at the LGM compared to modern, and stomatal conductance was higher by 60%, consistent with the general pattern shown by Becklin *et al*.^39^. GPP was reduced by 24% and NPP was reduced by 22% due to the low c_i_. However, the allocation to foliage increased from 18.2% at high CO_2_ to 20.8% at low CO_2_ while the allocation to roots decreased from 16.5% to 14.2%.

**Figure 5:**
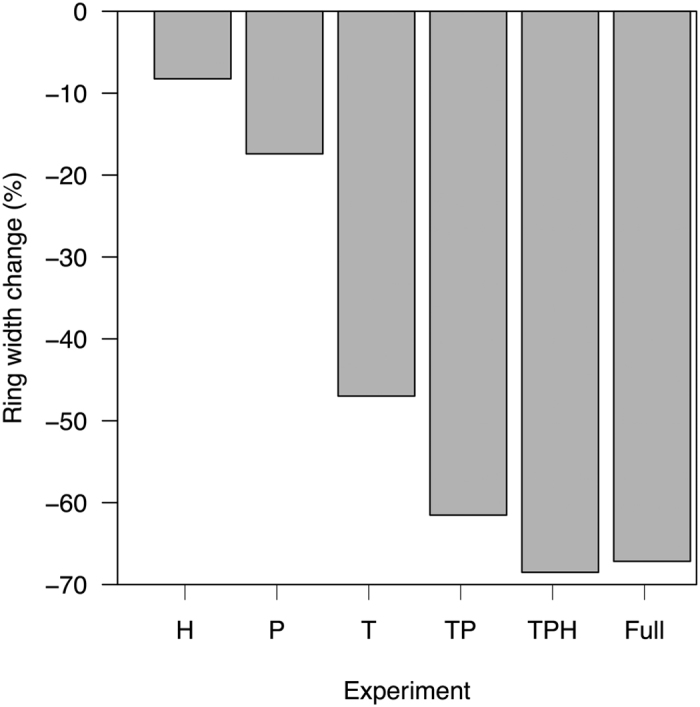
Impact of individual climate variables on simulated glacial ring widths. Each experiment is run by imposing modern values for a variable or combination of variables with all other variables (including CO_2_) held at glacial values. The bars show the percentage change in simulated ring width between each experiment and the baseline glacial simulation. H has modern relative humidity; P has modern precipitation; T has modern temperature; TP has both modern temperature and precipitation; TPH has modern temperature, precipitation and relative humidity. In the final experiment (Full) all of the climate variables are modern and only CO_2_ is set to glacial levels.

The simulated reduction in GPP already takes account of one impact of the colder glacial climate on primary production through the reduction of photorespiration at lower temperatures: the carbon cost through photorespiration would be ~20% more without this reduction. However, temperature also affects moisture supply through reducing saturated atmospheric vapour pressure and hence evapotranspiration. The reduction in temperature and increase in precipitation, and the implied increase in relative humidity, at the LGM in this region all have favourable impacts on moisture supply – which could be of importance given the very large simulated reduction in root mass at the LGM. To examine the relative importance of each of these factors on radial growth at the LGM, we ran a series of sensitivity tests in which we substituted modern values of these variables separately and in combination (Fig. 5), holding all other variables (including [CO_2_]) at glacial values. When temperature is set to modern values, ring widths decrease by 47%. The effect of precipitation changes alone is smaller, leading to a reduction of ring widths by 17%. The combined impact of warmer temperature, less precipitation and decreased humidity is equivalent to a 68% decrease in ring width. These experiments suggest that regional changes in climate during the LGM contributed to the maintenance of tree growth under low [CO_2_].

## Discussion

Natural changes in [CO_2_], and indeed climate, since the Last Glacial Maximum were as large as (although much slower than) the projected changes over the 21st century as a result of anthropogenic changes in greenhouse gas concentrations^40^. Measurements on glacial fossil *Juniperus* wood provide a picture of tree growth after long-term adaptation to low [CO_2_] and glacial climate. The low temperature and low [CO_2_] during the glacial climate is the converse of modern changes towards high [CO_2_] and temperature and so we would expect biotic responses also to be opposite to those taking place today. Free-air Carbon dioxide Enrichment (FACE) experiments have shown increased root system biomass as a response to elevated [CO_2_]^14^,^15^,^16^,^17^,^18^,^19^,^41^. Increased carbon export to mycorrhizae or the rhizosphere could also be responsible for the increasing below ground allocation^20^,^42^. Either way, the response is presumably adaptive, reflecting the need for plants to acquire additional nutrients, most notably nitrogen, to support increased total net primary production that is made possible by higher photosynthetic rates under enhanced [CO_2_].

[CO_2_] has increased greatly since pre-industrial time (from 280 to 400 ppm). Increased photosynthesis at the leaf level is a nearly universal response to enhanced [CO_2_] in C_3_ plants^43^,^44^, as is the enhancement of water use efficiency, due to increased photosynthesis combined with reduced stomatal conductance, which has been calculated from stable carbon isotope measurements in tropical forests^24^ and elsewhere^23^. However, several studies including that of van der Sleen *et al*.^24^ have failed to show any concomitant increase in stem radial growth. A possible explanation mooted by van der Sleen *et al*. is increased below-ground carbon allocation^24^. The lack of a [CO_2_] signal in the radial growth of trees has also been noted consistently in mid- to high-latitude regions^22^,^23^. Model simulations for *Callitris* in the Great Western Woodlands, Western Australia, where the trees also show no increase in ring width with increasing [CO_2_], showed that a modest increase in the ratio of root mass to foliage area (ζ) is sufficient to account for the lack of a [CO_2_] signal in ring widths^30^. The phenomenon could not be explained by variation in any other parameter of the T model. Thus, it is plausible that allocation shifts – consistent with the increased nutrient requirements for growth under high [CO_2_] and reduced requirements under low [CO_2_] – could be causing an apparent homoeostasis in the radial growth rates of trees with respect to [CO_2_]. The homoeostasis in radial growth seen at La Brea is also apparent in other tree-ring records dated to the last glaciation^45^,^46^,^47^, suggesting that changes in allocation are a widespread response to both glacial-interglacial and contemporary CO_2_ changes.

Our model-based analysis indicates that the observed stability in tree ring widths through time is not necessarily indicative of neutral growth responses to changing climate and [CO_2_] but could plausibly be explained by changes in allocation. It has also been suggested that this stability reflects changes in nutrient (specifically nitrogen) availability from glacial to present^39^,^48^. Becklin *et al*.^39^ measured the nitrogen content of fossil leaf specimens from the LGM and the late Holocene, and argued that the observed declining leaf nitrogen levels would result in reduced photosynthetic capacity and hence growth as [CO_2_] increased^39^. Changes in allocation and in nutrient availability are not mutually exclusive hypotheses, however, since nutrient limitation would be an important mechanism driving enhanced below-ground allocation. More information about leaf^39^ and soil^48^ nitrogen content could help in separating the direct (e.g. growth effects) versus indirect effects (e.g. allocation responses) of increasing carbon availability and decreasing nitrogen status through time, but unfortunately such measurements are not available for the La Brea site. Thus, we have focused here on the response to environmental factors for which there is unequivocal evidence for local changes, specifically climate and [CO_2_]. The potential for changes in above- and below-ground allocation in response to any environmental changes (including nutrient availability) has profound implications for global change biology, physiological ecology, evolutionary biology, dendrochronology and dendroclimatology, and deserves more attention.

## Methods

### J*uniperus* Sampling

*Juniperus* tree specimens were obtained from the Rancho La Brea tar pits (Los Angeles, CA) and ^14^C dated to 22–55 ka BP. Modern *Juniperus* tree specimens from southern California were obtained by coring trees currently growing in the Angeles and San Bernardino National Forests, CA. Details of sampling locations and stable isotope analysis are provided in Gerhart *et al*.^4^. Samples were first cleaned of tar (for the glacial specimens), then sanded. Samples were then imaged at high resolution using a flatbed scanner and ring widths were measured to the nearest 0.1 mm using Photoshop. Measured c_i_/c_a_ and ring width on glacial samples from La Brea and modern sites from southern California are given in Supplementary Table 2.

### The PT model

Potential gross primary production (GPP) was simulated by a generic light-use-efficiency model (a modified version of the P model^27^,^28^). The resulting GPP is allocated to foliage, transport tissue, and fine-root production and turnover via a geometric tree-growth model (T model^29^) that has a limited number of species-specific parameters influencing the allocation of carbon to different compartments. Tree growth (including stem radial growth) is simulated on an annual basis.

Potential GPP is determined by the photosynthetically active radiation (PAR) incident on the vegetation canopy during the growing season (here defined as the period with temperatures above −1 °C, consistent with observations that photosynthesis can occur at temperatures below 0 °C in temperate climates^49^) determined by the maximum quantum efficiency of photosynthesis (Φ_0_), and the CO_2_ limitation effect:


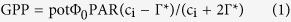


where Φ_0_ is set to 0.816 g C mol^−1^ photon, equivalent to a quantum efficiency of 0.085 mol C mol^−1^ photon^27^,^50^ and a leaf absorptance of 0.8 is assumed. PAR is calculated based on solar geometry and is subsequently modified by atmospheric transmission, depending on elevation and cloud cover.

The effects of photorespiration and substrate limitation at subsaturating [CO_2_] are represented as a function of the leaf-internal [CO_2_] (c_i_) and the photorespiratory compensation point (Γ*). c_i_ is obtained from


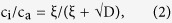


where D is the vapour pressure deficit, VPD, and ξ is the stomatal sensitivity. ξ is obtained from:


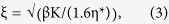


where β is the ratio of unit costs for carboxylation and transpiration at 25 °C; Κ is the effective Michaelis–Menten coefficient for Rubisco-limited photosynthesis, depending on temperature, atmospheric pressure and oxygen concentration; η* is the viscosity correction factor (the ratio of the viscosity of water at current temperature to that at 25 °C).

The photorespiratory compensation point (Γ*) is controlled by temperature, and described by an exponential closely approximating the Arrhenius function:


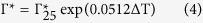


where Γ*_25_ is the value of Γ* at 25 °C (4.331 Pa), and ΔT is the temperature difference from 25 °C.

GPP is further modified by a factor (α/1.26)^1/4^ (α is the ratio of actual to equilibrium evapotranspiration) to account for GPP reduction due to drought.

GPP from the P model is used as input to the T model. In this model, the fraction of incident PAR absorbed by the canopy (fAPAR) is estimated from the leaf area index (LAI) within the canopy and used to convert potential to actual GPP using Beer’s law. Annual net primary production (NPP) is derived from annual GPP, corrected for foliage respiration, by deducting growth respiration (assumed to be proportional to NPP) and the maintenance respiration of sapwood and fine roots. NPP is allocated to stem, foliage and fine-root increments, foliage turnover and fine-root turnover. Carbon is allocated to different tissues within the constraint of the basic functional or geometric relationships between different dimensions of the tree, including asymptotic height-diameter trajectories. A full description of the T model is given in Li *et al*.^29^. The P and T model codes are available from (https://guangqili@bitbucket.org/guangqili/pt-model.git).

The T model requires 11 species-specific parameters to be specified, most of which can be obtained from the forestry literature (Supplementary Table 1). The shape parameters (initial slope of the height-diameter relationship, initial ratio of crown area to stem area, maximum tree height) are site specific, but were not available for either the CA640 or the La Brea trees. Parameter values for sapwood specific respiration and the ratio of fine root mass to foliage area for *Juniperus* were not available. Appropriate values for the shape parameters for *Juniperus*, and generic needleleaf tree values for sapwood specific respiration and the ratio of fine root mass to foliage area, were derived from the literature (Supplementary Table 1) and then optimized using approximate Bayesian computation^36^,^37^,^38^ using observed climate and [CO_2_] for the period 1930–1980. The calibration objective was to minimise the absolute difference between modelled and observed mean tree-ring width at site CA640 (36.95°N, 118.92°W, 2630 m a.s.l.) during the calibration period and the criterion for convergence was a difference of no more than ±2.5% of the mean value. The prior of each parameter (Supplementary Table 1) was based on the median of the published values, and the standard deviation was set to half of the median value.

The T model accumulates carbon over an effective growing season, here set to 2 years commencing from the middle of the year two years before the year of tree-ring formation (July of −2 year to June of current year) following the treatment for water-limited species described in Li *et al*. (2015)^30^. We checked that this treatment was appropriate for *Juniperus* in California using an ordinary least-squares multiple linear regression of driving climate variables (total annual photosynthetically active radiation: PAR, mean annual temperature: MAT, the ratio of actual to potential evapotranspiration: α, vapour pressure deficit: VPD, and [CO_2_]) accumulated over varying periods of time against mean tree-ring width from CA640 during the period from 1930–1980. This analysis confirmed that the 2-year accumulation period was appropriate and correlations were significantly worse for either longer or shorter intervals.

### Climate and CO_2_ input: modern

Climate data for the CA640 site was obtained from 0.5°CRU TS v3.22 data set^51^, which provides time series of mean monthly temperature, diurnal temperature range, precipitation, vapour pressure and cloudiness (CRU: https://crudata.uea.ac.uk/cru/data/hrg/). It is not possible to discriminate the elevations of the modern southern Californian sites at this resolution. We therefore derived an elevational correction factor for each monthly climate variable using the modern climatology data from the 10′ CRU CL 2.0^52^ and applied this to the time series data to derive interannually-varying climate values at the elevation of each site. Modern [CO_2_] observations are based on merging ice-core records for the interval from 1901 to 1957^53^,^54^ and the yearly average of direct atmospheric measurements from Mauna Loa and the South Pole stations from 1958 to 2013 (http://scrippsco2.ucsd.edu/data/merged_ice_core/merged_ice_core_yearly.csv).

### Climate and CO_2_ input: LGM

We used model outputs from a Last Glacial Maximum (LGM, 21,000 years ago) and a control simulation (piControl) run with the Community Climate System Model Version 4 (CCSM4) following the PMIP3/CMIP5 protocol (https://pmip3.lsce.ipsl.fr/). The LGM simulations are available from the Earth System Grid Federation (ESGF: http://cmippcmdi.llnl.gov/cmip5/data_getting_started.html). This is an equilibrium simulation with fixed values of CO_2_ throughout (180 ppm) and thus the interannual variability is not forced but reflects internal variability. The simulated times series of LGM monthly climate was corrected, to remove the potential difference between the piControl and the modern climate at each site, by adding the difference between the long term mean of the last 100 years of the piControl and the elevation-adjusted climatology between 1961–1990 at each site to the LGM times series. Glacial [CO_2_] are from the Taylor Dome ice-core records^55^.

### Simulations

The PT model was run using inputs of monthly climate and annual changes in CO_2_. Ring widths for CA640 are available from the International Tree Ring Database (https://www.ncdc.noaa.gov/data-access/paleoclimatology-data/datasets/tree-ring). Information about the diameter of the modern trees used to construct the CA640 record at the time of sampling is not available. We therefore initialized the T model component of the simulations with a diameter of 0.3 m. The simulations were run with varying climate and CO_2_ from 1903–1985. Model outputs of simulated ring width for the modern validation are given in Supplementary Table 3.

The calculations of simulated c_i_/c_a_ under modern day conditions, at the elevation of the La Brea Tar Pit and for each of the southern California sites, were made for the period of 1951–2000. This interval was chosen to maximize comparability with the observed values of c_i_/c_a_ at the southern Californian sites. The calculation of simulated c_i_/c_a_ under glacial conditions was made using the last 100 years of the LGM simulation. Given that this is an equilibrium simulation, the different length of the averaging period between LGM and modern makes no difference to the comparison. In addition to the LGM simulation, we ran two sensitivity tests in which we used (a) modern monthly temperatures with glacial monthly VPD and (b) glacial monthly temperatures with modern monthly VPD. These are the only two climate variables that impact the calculation of c_i_/c_a_ in the P model. The impact of the small change in elevation above sea level between LGM and present (~120 m) is negligible for these calculations.

Tree growth under LGM conditions was simulated using the parameters values used for the modern simulations, with the exception of the leaf area index and the ratio of fine root mass to foliage area. These allocation parameters were optimized using approximate Bayesian computation^36^,^37^,^38^ using simulated LGM climate and 180 ppm [CO_2_]. The calibration objective was to minimise the absolute difference between modelled and observed mean tree-ring width (1.83 mm at 22 ka) and the criterion for convergence was a difference of no more than ±2.5% of the mean value.

The simulations to test the sensitivity of radial growth to individual climate variables were run by perturbing monthly temperature, precipitation and relative humidity singly and in combination. In each test, the modern values of the variable were substituted for the glacial values, holding all other variables including [CO_2_] at glacial values. Model outputs of simulated GPP, NPP, c_i_/c_a_, ring width, V_cmax_, stomatal conductance (g_s_) for modern and LGM simulations at La Brea, and for the LGM sensitivity runs are given in Supplementary Table 4.

## Additional Information

**How to cite this article**: Li, G. *et al*. Changes in biomass allocation buffer low CO_2_ effects on tree growth during the last glaciation. *Sci. Rep.* **7**, 43087; doi: 10.1038/srep43087 (2017).

**Publisher's note:** Springer Nature remains neutral with regard to jurisdictional claims in published maps and institutional affiliations.

## Supplementary Material

Supplementary Material

Supplementary Dataset 2

Supplementary Dataset 3

Supplementary Dataset 4

## References

[b1] NGRIP Project Members. High-resolution record of Northern Hemisphere climate extending into the last interglacial period. Nature 431, 147–151 (2004). 15356621 10.1038/nature02805

[b2] EPICA Community Members. Eight glacial cycles from an Antarctic ice core. Nature 429, 623–628 (2004). 15190344 10.1038/nature02599

[b3] Ward J. K. . Carbon starvation in glacial trees recovered from the La Brea tar pits, southern California. Proceedings of the National Academy of Sciences of the United States of America 102, 690–694 (2005). 15642948 10.1073/pnas.0408315102 PMC544040

[b4] Gerhart L. M., Harris J. M., Nippert J. B., Sandquist D. R. & Ward J. K. Glacial trees from the La Brea tar pits show physiological constraints of low CO_2_. New Phytologist 194, 63–69 (2012). 22187970 10.1111/j.1469-8137.2011.04025.x

[b5] Gerhart L. M. Glacial Tree Physiology: Using Stable Isotopes to Reconstruct Plant Responses to Environmental Change Since the Last Glacial Period Ph.D. thesis, University of Kansas (2013).

[b6] Thompson R. S., Whitlock C., Bartlein P. J., Harrison S. P. & Spaulding W. G. Climatic changes in the western United States since 18,000 yr BP In Global Climates since the Last Glacial Maximum (eds Wright H. E. Jr., Kutzbach J. E., Webb T. III., Ruddiman W. F., Street-Perrott F. A. & Bartlein P. J.) 468–513 (University of Minnesota Press, Minneapolis, 1993).

[b7] Clark P. U. . The Last Glacial Maximum. Science 325, 710–714 (2009). 19661421 10.1126/science.1172873

[b8] Bartlein P. J. . Pollen-based continental climate reconstructions at 6 and 21 ka: a global synthesis. Climate Dynamics 37, 775–802 (2011).

[b9] COHMAP Members. Climatic changes of the last 18,000 years: observations and model simulations. Science 241, 1043–1052 (1988). 17747487 10.1126/science.241.4869.1043

[b10] Braconnot P. . Results of PMIP2 coupled simulations of the mid-Holocene and Last Glacial Maximum, Part 1: experiments and large-scale features. Climate of the Past 3, 261–277 (2007).

[b11] Ullman D. J., LeGrande A. N., Carlson A. E., Anslow F. S. & Licciardi J. M. Assessing the impact of Laurentide Ice Sheet topography on glacial climate. Climate of the Past 10, 487–507 (2014).

[b12] Ward J. K., Myers D. A. & Thomas R. B. Physiological and growth responses of C_3_ and C_4_ plants to reduced temperature when grown at low CO_2_ of the last ice age. Journal of Integrative Plant Biology 50, 1388–1395 (2008). 19017126 10.1111/j.1744-7909.2008.00753.x

[b13] Gerhart L. M. & Ward J. K. Plant responses to low [CO_2_] of the past. New Phytologist 188, 674–695 (2010). 20840509 10.1111/j.1469-8137.2010.03441.x

[b14] Norby R. J., Ledford J., Reilly C. D., Miller N. E. & O’Neill E. G. Fine-root production dominates response of a deciduous forest to atmospheric CO_2_ enrichment. Proceedings of the National Academy of Sciences of the United States of America 101, 9689–9693 (2004). 15210962 10.1073/pnas.0403491101 PMC470736

[b15] DeLucia E. H. . Net primary production of a forest ecosystem with experimental CO_2_ enrichment. Science 284, 1177–1179 (1999). 10325230 10.1126/science.284.5417.1177

[b16] Pritchard S. G. . Fine root dynamics in a loblolly pine forest are influenced by free-air-CO_2_-enrichment: A six_-_year-minirhizotron study. Global Change Biology 14, 588–602 (2008).

[b17] King J. . Fine-root biomass and fluxes of soil carbon in young stands of paper birch and trembling aspen as affected by elevated atmospheric CO_2_ and tropospheric O_3_. Oecologia 128, 237–250 (2001). 10.1007/s004420100656 28547473

[b18] Calfapietra C. . Free-air CO_2_ enrichment (FACE) enhances biomass production in a short-rotation poplar plantation. Tree Physiology 23, 805–814 (2003). 12865246 10.1093/treephys/23.12.805

[b19] Lukac M., Calfapietra C. & Godbold D. L. Production, turnover and mycorrhizal colonization of root systems of three *Populus* species grown under elevated CO_2_ (POPFACE). Global Change Biology 9, 838–848 (2003).

[b20] Godbold D. L. . Elevated atmospheric CO_2_ affects ectomycorrhizal species abundance and increases sporocarp production under field conditions. Forests 6, 1256–1273 (2015).

[b21] Battipaglia G. . Elevated CO_2_ increases tree‐level intrinsic water use efficiency: insights from carbon and oxygen isotope analyses in tree rings across three forest FACE sites. New Phytologist 197, 544–554 (2013). 23215904 10.1111/nph.12044

[b22] Gedalof Z. E. & Berg A. A. Tree ring evidence for limited direct CO2 fertilization of forests over the 20th century. Global Biogeochemical Cycles 24, GB3027, doi: 10.1029/2009GB003699 (2010).

[b23] Andreu-Hayles L. . Long tree‐ring chronologies reveal 20th century increases in water‐use efficiency but no enhancement of tree growth at five Iberian pine forests. Global Change Biology 17, 2095–2112 (2011).

[b24] van der Sleen P. . No growth stimulation of tropical trees by 150 years of CO_2_ fertilization but water-use efficiency increased. Nature Geoscience 8, 24–28 (2015).

[b25] Temme A., Cornwell W., Cornelissen J. & Aerts R. Meta‐analysis reveals profound responses of plant traits to glacial CO_2_ levels. Ecology and Evolution 3, 4525–4535 (2013). 24340192 10.1002/ece3.836 PMC3856751

[b26] Mohan J. E., Clark J. S. & Schlesinger W. H. Genetic variation in germination, growth, and survivorship of red maple in response to subambient through elevated atmospheric CO_2_. Global Change Biology 10, 233–247 (2004).

[b27] Wang H., Prentice I. C. & Davis T. Biophysical constraints on gross primary production by the terrestrial biosphere. Biogeosciences 11, 5987–6001 (2014).

[b28] Wang H. . A universal model for carbon dioxide uptake by plants. bioRxiv 040246, 10.1101/040246 (2016). 29150690

[b29] Li G., Harrison S. P., Prentice I. C. & Falster D. Simulation of tree-ring widths with a model for primary production, carbon allocation, and growth. Biogeosciences 11, 6711–6724 (2014).

[b30] Li G., Harrison S. P. & Prentice I. C. A model analysis of climate and CO_2_ controls on tree growth in a semi-arid woodland. Ecological Modelling 342, 175–185 (2016).

[b31] Graumlich L. J. A 1000-year record of temperature and precipitation in the Sierra Nevada. Quaternary Research 39, 249–255 (1993).

[b32] Wright I. J. & Westoby M. Leaves at low versus high rainfall: coordination of structure, lifespan and physiology. New Phytologist 155, 403–416 (2002). 10.1046/j.1469-8137.2002.00479.x 33873314

[b33] Prentice I. C., Dong N., Gleason S. M., Maire V. & Wright I. J. Balancing the costs of carbon gain and water transport: testing a new theoretical framework for plant functional ecology. Ecology Letters 17, 82–91 (2014). 24215231 10.1111/ele.12211

[b34] Gent P. R. . The Community Climate System Model Version 4. Journal of Climate 24, 4973–4991 (2011).

[b35] Brady E. C., Otto-Bliesner B. L., Kay J. E. & Rosenbloom N. Sensitivity to glacial forcing in the CCSM4. Journal of Climate 26, 1901–1925 (2013).

[b36] Jaakkola T. S. & Jordan M. I. Bayesian parameter estimation via variational methods. Statistics and Computing 10, 25–37 (2000).

[b37] Pelikan M. Bayesian optimisation algorithm. Studies in Fuzziness and Soft Computing 170, 31–48 (2005).

[b38] van der Vaart E., Beaumont M. A., Johnston A. S. & Sibly R. M. Calibration and evaluation of individual-based models using Approximate Bayesian Computation. Ecological Modelling 312, 182–190 (2015).

[b39] Becklin K. M., Medeiros J. S., Sale K. R. & Ward J. K. Evolutionary history underlies plant physiological responses to global change since the last glacial maximum. Ecology Letters 17, 691–699 (2014). 24636555 10.1111/ele.12271 PMC4097002

[b40] Braconnot P. . Evaluation of climate models using palaeoclimatic data. Nature Climate Change 2, 417–424 (2012).

[b41] Smith A. R., Lukac M., Bambrick M., Miglietta F. & Godbold D. L. Tree species diversity interacts with elevated CO_2_ to induce a greater root system response. Global Change Biology 19, 217–228 (2013). 23504733 10.1111/gcb.12039

[b42] Phillips D. L., Johnson M. G., Tingey D. T., Catricala C. E., Hoyman T. L. & Nowak R. S. Effects of elevated CO_2_ on fine root dynamics in a Mojave Desert community: a FACE study. Global Change Biology 12, 61–73 (2006).

[b43] Ainsworth E. A. & Rogers A. The response of photosynthesis and stomatal conductance to rising [CO_2_]: mechanisms and environmental interactions. Plant, Cell & Environment 30, 258–270 (2007). 10.1111/j.1365-3040.2007.01641.x 17263773

[b44] Pinto H., Sharwood R. E., Tissue D. T. & Ghannoum O. Photosynthesis of C_3_, C_3_–C_4_, and C_4_ grasses at glacial CO_2_. Journal of Experimental Botany 65, 3669–3681 (2014). 24723409 10.1093/jxb/eru155 PMC4085965

[b45] Roig F. A. . Climate variability 50,000 years ago in mid-latitude Chile as reconstructed from tree rings. Nature 410, 567–570 (2001). 11279491 10.1038/35069040

[b46] Takahashi H. A., Yonenobu H., Nakamura T. & Wada H. Japanese cypress tree rings from the last glacial period – possibility of palaeoenvironment reconstruction. Radiocarbon 143, 433–438 (2001).

[b47] Palmer J. . Extension of New Zealand kauri (*Agathis australis*) tree-ring chronologies into Oxygen Isotope Stage (OIS) 3. Journal of Quaternary Science 21, 779–787 (2006).

[b48] McLauchlan K. K., Williams J. J., Craine J. M. & Jeffers E. S. Changes in global nitrogen cycling during the Holocene epoch. Nature 495, 352–355 (2013). 23518563 10.1038/nature11916

[b49] Larcher W. Physiological Plant Ecology: Ecophysiology and Stress Physiology of Functional Groups. (Springer, 2003).

[b50] Collatz G. J., Berry J. A. & Clark J. S. Effects of climate and atmospheric CO_2_ partial pressure on the global distribution of C_4_ grasses: present, past, and future. Oecologia 114, 441–454 (1998). 10.1007/s004420050468 28307893

[b51] Harris I., Jones P., Osborn T. & Lister D. Updated high‐resolution grids of monthly climatic observations–the CRU TS3.10 dataset. International Journal of Climatology 34, 623–642 (2014).

[b52] New M., Lister D., Hulme M. & Makin I. A high-resolution data set of surface climate over global land areas. Climate Research 21, 1–25 (2002).

[b53] Etheridge D. . Natural and anthropogenic changes in atmospheric CO_2_ over the last 1000 years from air in Antarctic ice and firn. Journal of Geophysical Research: Atmospheres 101, 4115–4128 (1996).

[b54] MacFarling Meure C. . Law Dome CO_2_, CH_4_ and N_2_O ice core records extended to 2000 years BP. Geophysical Research Letters 33, L14810, doi: 10.1029/2006GL026152 (2006).

[b55] Indermühle A., Monnin E., Stauffer B., Stocker T. F. & Wahlen M. Atmospheric CO_2_ concentration from 60 to 20 kyr BP from the Taylor Dome ice core, Antarctica. Geophysical Research Letters 27, 735–738 (2000).

