# Supplementary material for: Changes in biomass allocation buffer low CO2 effects on tree growth during the last glaciation
Source: Sci Rep. 2017 Feb 24;7:43087. doi: 10.1038/srep43087 (PMC5324044; doi:10.1038/srep43087)
Supplement: Supplementary Material [file srep43087-s1.pdf]

Supplementary Material

Changes in biomass allocation buffer low CO_2 effects on tree growth during the last glaciation

Guangqi Li[1,2], Laci M. Gerhart[3], Sandy P. Harrison[1,2], Joy K. Ward[4], John M. Harris[5] & I. Colin Prentice[1,6]

1 Department of Biological Sciences, Macquarie University, North Ryde, NSW 2109, Australia

2 School of Archaeology, Geography and Environmental Sciences (SAGES), Reading University, Reading, UK

3 Geography Department, Kansas State University, Manhattan, KS 66505, USA

4 Department of Ecology and Evolutionary Biology, University of Kansas, Lawrence, KS 66045, USA

5 Curator Emeritus, The La Brea Tar Pits Museum (George C. Page Museum), 5801 Wilshire Boulevard, Los Angeles, CA 90036, USA

6 AXA Chair of Biosphere and Climate Impacts, Department of Life Sciences, Imperial College London, Silwood Park Campus, Buckhurst Road, Ascot SL5 7PY, UK

This supplementary contains tables describing model parameter values (Supplementary Table 1), tree-ring observations from La Brea (Supplementary Table 2), modern observed and simulated tree rings at the CA640 site (Supplementary Table 3) and outputs from the modern and LGM simulations for La Brea (Supplementary Table 4). It also includes a figure showing the relationship between tree ring width and individual climate parameters (Supplementary Fig. 1).

Supplementary Fig 1. Simulated and observed response of tree radial growth at site CA640 to climate and [CO_2]. Partial residual plots based on the regression analysis, obtained using the visreg package in R, are shown. The dependent variable is mean ring width (from 1930 to 1980). The predictor variables are total incident photosynthetically active radiation (PAR_0), mean annual temperature during growing season, vapour pressure deficit (VPD), the ratio of actual to potential evapotranspiration (α), and annual [CO_2]. The analyses shown were performed on the 50-year interval 1930-1980 because the climate data for the pre-1930 period is less reliable. However, the form of the relationships between ring width and individual climate variables and [CO_2] are the same when the analyses are run for the longer period (1903-1985) covered by the CA640 record.

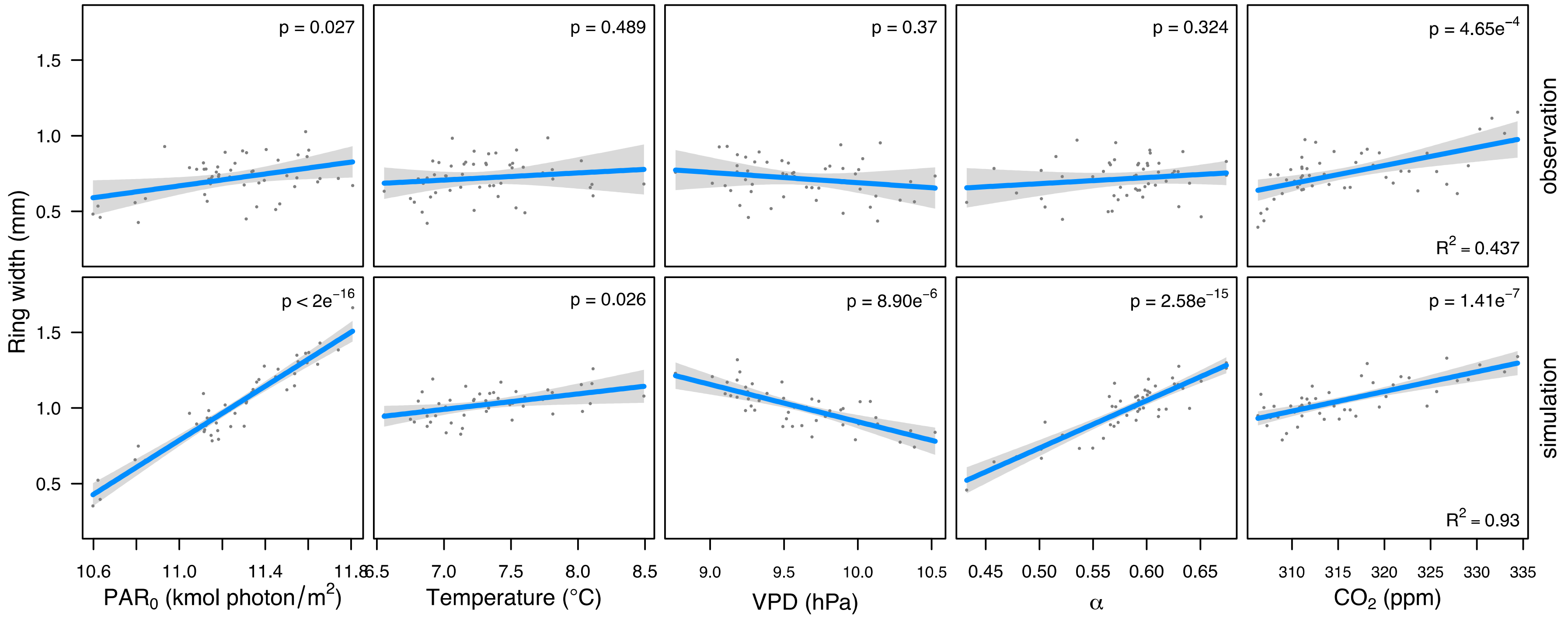

Supplementary Table 1 Bayesian parameter optimisation for *Juniperus* in site CA640 (36.95°N, 118.92°W, 2630 m a.s.l.) under modern climate and [CO_2], and in La Brea (34.06ºN, 118.36ºW, 80 m.a.s.l.) under LGM climate and 180 ppm [CO_2]. Superscripts in column 3 refer to publications in the reference list below.

Parameter	Symbol	Uncertainty or range of values from literature	Source of information for CA640 simulation	CA640			La Brea (180ppm)		
				Prior value	Posterior value	Accepted value	Prior value	Posterior value	Accepted value
initial slope of height-diameter relationship (–)	a	-	Bayesian parameter optimization	60±30	82.58±17.47	82.58			82.58
initial ratio of crown area to stem cross-sectional area (–)	c	-	Bayesian parameter optimization	500±250	445.69±222.14	445.69			445.69
maximum tree height (m)	H_m	-	Bayesian parameter optimization	12±6	12.38±4.90	12.38			12.38
sapwood density (kg C m^{-3})	ρ_s	250	*Juniperus spp.*			250			250
specific leaf area (m^2 kg^{-1} C)	σ	6.54 ~ 7.76[1], 5.6 ~ 6.54[1]	*Juniperus ashei*, *Juniperus erythrocarpa*			6.54			6.54
foliage turnover time (yr)	τ_f	6.5, 8.2[2]	*Juniperus monosperma*			7			7
fine-root turnover time (yr)	τ_r	0.76±0.06[3]	*Juniperus osteosperma*			1			1
fine-root specific respiration rate (yr^{-1})	r_r	1.36[4]	*Juniperus monosperma*			1.36			1.36
sapwood specific respiration rate (yr^{-1})	r_s	0.5~10 (20)[5]	Bayesian parameter optimization	1±0.5	1.03±0.44	1.03			1.03
leaf area index within the crown (–)	L	1.5~12.5[1]	*Juniperus erythrocarpa*			6	6±3	5.38±2.92	5.38
ratio of fine-root mass to foliage area (kgC m^{-2})	ζ	0.17[6]	Bayesian parameter optimization	0.10±0.15	0.17±0.02	0.17	0.10±0.15	0.12±0.06	0.12

Supplementary Table 2 Measurements of c_i/c_a and ring width. Observations include measurements of ring width and c_i:c_a for fossil wood specimens from ca 22 ka BP from La Brea tar pits and living trees at six nearby sites. Data for other glacial age specimens from la Brea, which were not used in the current modelling, are given in Ward et al. (2005) and Gerhart et al. (2012).

Supplementary Table 3 Simulated and observed *Juniperus occidentalis* ring width for the period 1903 to 1985, from site CA640 (36.95ºN, 118.92ºW, 2630 m. a.s.l.). PT model was driven by modern climate and [CO_2], and was initialised with a standard tree (DBH=0.3m).

Supplementary Table 4 Simulated c_i/c_a, ring width, GPP, NPP, V_{cmax}, g_s for modern and LGM simulations at La Brea and the 6 modern southern California sites. Models are driven by the following conditions at the La Brea tar pits site: 1) LGM full climate and 180 ppm [CO_2] (LGM), 2) modern full climate and transient [CO_2] (Modern), 3) LGM full climate and 320 ppm [CO_2] (LGM+320), 4) LGM climate except with the substitution of modern humidity at 180 ppm [CO_2] (LGM+H), 5) LGM climate except with the substitution of modern precipitation at 180 ppm [CO_2] (LGM+P), 6) LGM climate except with the substitution of modern temperature at 180 ppm [CO_2] (LGM+T), 7) LGM climate except with the substitution of modern temperature and precipitation at 180 ppm [CO_2] (LGM+TP), 8) LGM climate except with the substitution of modern temperature, precipitation and humidity at 180 ppm [CO_2] (LGM+TPH), 9) LGM climate except with the substitution of modern VPD at 180 ppm [CO_2] (LGM+TPH), 10) modern climate with 180 ppm [CO_2] (MOD+180). For the 6 modern southern California sites, the model was run with 4 climates (LGM, Modern, LGM+T, and LGM+VPD). The simulations are all initialized with a standard tree (DBH=0.3m).

Supplementary references

1 Owens, M. & Ansley, J. Ecophysiology and growth of Ashe and red berry juniper. In *Juniper Symposium*, Technical Report 97-1, 19-31 (Texas A&M University: San Angelo, Texas, 1997).

2 Reich, P. B. *et al.* Generality of leaf trait relationships: a test across six biomes. *Ecology* **80**, 1955-1969 (1999).

3 Peek, M. S. *et al.* Root turnover and relocation in the soil profile in response to seasonal soil water variation in a natural stand of Utah juniper (*Juniperus osteosperma*). *Tree Physiology* **26**, 1469-1476 (2006).

4 Burton, A., Pregitzer, K., Ruess, R., Hendrick, R. & Allen, M. Root respiration in North American forests: effects of nitrogen concentration and temperature across biomes. *Oecologia* **131**, 559-568 (2002).

5 Landsberg, J. J. & Sands, P. *Physiological Ecology of Forest Production: Principles, Processes and Models*. (Academic Press, London, 2010).

6 White, M. A., Thornton, P. E., Running, S. W. & Nemani, R. R. Parameterization and sensitivity analysis of the BIOME-BGC terrestrial ecosystem model: net primary production controls. *Earth Interactions* **4**, 1-85 (2000).
